# Microfluidic Formation of Honeycomb-Patterned Droplets Bounded by Interface Bilayers via Bimodal Molecular Adsorption

**DOI:** 10.3390/mi11070701

**Published:** 2020-07-20

**Authors:** Shougo Fujiwara, Kan Shoji, Chiho Watanabe, Ryuji Kawano, Miho Yanagisawa

**Affiliations:** 1 Komaba Institute for Science, The University of Tokyo, Komaba 3-8-1, Meguro, Tokyo 153-8902, Japan; fujiwara.3132.10@gmail.com (S.F.); chiho.watanabe@g.ecc.u-tokyo.ac.jp (C.W.); 2 Department of Applied Physics, Tokyo University of Agriculture and Technology, Naka-cho 2-24-16, Koganei, Tokyo 184-8588, Japan; 3 Department of Biotechnology and Life Science., Tokyo University of Agriculture and Technology, Naka-cho 2-24-16, Koganei, Tokyo 184-8588, Japan; kshoji@mech.nagaokaut.ac.jp (K.S.); rjkawano@cc.tuat.ac.jp (R.K.); 4 Department of Mechanical Engineering, Nagaoka University of Technology, Kamitomioka 1603-1, Nagaoka, Niigata 940-2188, Japan; 5 Department of Basic Science, The University of Tokyo, Komaba 3-8-1, Meguro, Tokyo 153-8902, Japan

**Keywords:** self-assembly, microfluidic device, lipid bilayers, droplet adhesion, close packed pattern

## Abstract

Assembled water-in-oil droplets bounded by lipid bilayers are used in synthetic biology as minimal models of cell tissue. Microfluidic devices successfully generate monodispersed droplets and assemble them via droplet interface bilayesr (DIB) formation. However, a honeycomb pattern of DIB-bounded droplets, similar to epithelial tissues, remains unrealized because the rapid DIB formation between the droplets hinders their ability to form the honeycomb pattern. In this paper, we demonstrate the microfluidic formation of a honeycomb pattern of DIB-bounded droplets using two surfactants with different adsorption rates on the droplet surface. A non-DIB forming surfactant (sorbitan monooleate, Span 80) was mixed with a lipid (1,2-dioleoyl-sn-glycero-3-phosphocholine, PC), whose adsorption rate on the droplet surface and saturated interfacial tension were lower than those of Span 80. By changing the surfactant composition, we established the conditions under which the droplets initially form a honeycomb pattern and subsequently adhere to each other via DIB formation to minimize the interfacial energy. In addition, the reconstituted membrane protein nanopores at the DIBs were able to transport molecules. This new method, using the difference in the adsorption rates of two surfactants, allows the formation of a honeycomb pattern of DIB-bounded droplets in a single step, and thus facilitates research using DIB-bounded droplet assemblies.

## 1. Introduction

Lipid bilayers formed between adhesive water-in-oil (W/O) droplets, called droplet interface bilayers (DIBs), are often used to model biomembranes [1,2,3,4]. A DIB is easily formed by putting two W/O droplets covered by a lipid monolayer into contact with each other. By reconstituting the membrane protein nanopores in the DIBs, the molecular transport among the droplets can be analyzed [4,5,6,7]. Therefore, DIB-bounded droplets have the potential to model cell tissues for analyzing the molecular transport in a cell-sized space; this is relatively difficult for adhered liposomes, which require electrostatic interactions [8,9,10,11,12,13] or synthesized adhesion molecules for preparation [14,15]. 

For DIB-bounded droplets to model cell tissues, numerous droplets need to be assembled into a honeycomb pattern because many epithelial tissues pack cells into a honeycomb pattern to support their structural and functional integrity [16]. Droplet-based microfluidics enable the preparation of numerous monodispersed droplets [17]. When droplets covered by a lipid layer are collected in a narrow channel, they adhere to each other and spontaneously form DIB-bounded droplets in a single step [2,3], similar to those prepared by manual assembly [5,6,17,18] or using three-dimensional printing technology [19,20]. However, to the best of our knowledge, formation of a honeycomb pattern of DIB-bounded droplets has yet to be achieved because the rapid DIB formation hinders their spatial rearrangement to form a honeycomb pattern [21,22,23]. If a honeycomb pattern of DIB-bounded droplets could be obtained, it can be used as a cell tissue model for the physicochemical understanding of intercellular transport and cellular geometry [19,20,24]. By changing the interior phase from a liquid to a gel, we could also regulate the mechanical properties of the cell tissue model to be more realistic [25]. 

**Figure 1:**
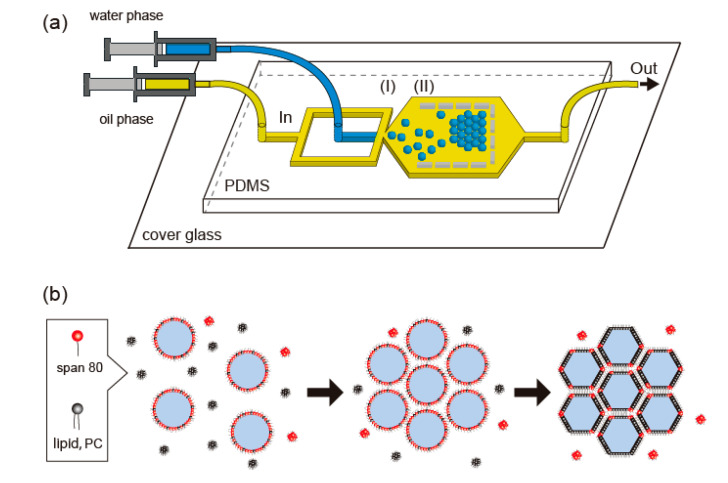
Schematic of (**a**) a microfluidic device for preparing the close-packed honeycomb pattern of assembled droplets in a two-dimensional space and of (**b**) droplet interface bilayer (DIB) formation among droplets. Span 80 molecules (red), having a higher adsorption rate than lipid 1,2-dioleoyl-sn-glycero-3-phosphocholine (PC,black), initially accumulate on droplet surface. Replacement of Span 80 with PC initiates DIB formation and surface adhesion among droplets.

Here, we demonstrate the honeycomb pattern formation of DIB-bounded droplets in a microfluidic device using two surfactants with different adsorption rates on the droplet surface (Figure 1a). Because the adsorption rate of a lipid on a droplet surface was reported to be quite low (more than a few minutes [26,27]), we propose the use of a surfactant, sorbitan monooleate (Span 80), which has a higher adsorption rate than a lipid (Figure 1b). We expected that Span 80 would initially accumulate on the droplet surface prior to the lipid, 1,2-dioleoyl-sn-glycero-3-phosphocholine (PC), and stabilize the droplets until they formed a honeycomb pattern in the microfluidic device. The replacement of Span 80 with PC meant that DIB formation would occur spontaneously due to the decrease in interfacial energy. By changing the composition between Span 80 and the lipid in oil phase, we identified a condition under which the droplets form a honeycomb pattern and subsequently undergo surface-to-surface contact with adjacent droplets via DIB formation. This method focuses on the adsorption rate and enables the formation of a honeycomb pattern of DIB-bounded droplets in a single step without manually assembling the droplets or changing the device design. Therefore, these results present new perspectives for DIB-bounded droplet assembly in a microfluidic device and facilitate studies on model cell tissues.

## 2. Materials and Methods

### 2.1. Materials 

A zwitterionic lipid, PC (Avanti Polar Lipids, Alabaster, AL, USA), and a nonionic surfactant, sorbitan monooleate (Span80, Tokyo Chemical Industry Co., Tokyo, Japan), were dissolved in hexadecane (purity > 98%; Tokyo Chemical Industry Co., Tokyo, Japan) and used as the continuous oil phase. Milli-Q water (H20MB0124, Merck KGaA) was used as the aqueous phase. To visualize a lipid membrane covering a droplet, a fluorescent-labeled lipid, 1,2-dimyristoyl-sn-glycero-3-phosphoethanolamine-N-(lissamine rhodamine B sulfonyl) (ammonium salt; Rho-PE, Avanti Polar Lipids) was used. Calcein (Sigma-AldrichSt. Louis, MO, USA), a water-soluble fluorescein, was used to analyze the function of α-hemolysin isolated from *Staphylococcus aureus* (Sigma-Aldrich) reconstituted in a DIB. The microfluidic device was developed using a photoresist (SU-8 3025, MicroChem Corp., Westborough, MA, USA) and a polydimethylsiloxane (PDMS) package (Sylgard 184, Dow Corning Toray Corp., Tokyo, Japan). 

### 2.2. Microfluidic Device for Droplet Formation and Droplet Assembly

We fabricated the microfluidic device as illustrated using standard photolithography processes [28,29] with a photoresist, SU-8 3025. Film photomasks were designed using two-dimensional computer-aided design (2D-CAD) software (AutoCAD 2014, AUTODESK, San Francisco, CA, USA) and fabricated with a laser spot diameter and drawing pitch of 4 and 1 μm, respectively. A patterned UV light was exposed using a UV exposure system (UV-KUB2, KLOE, Montpellier, France). A microchannel was fabricated by polydimethylsiloxane (PDMS) molding, in which the PDMS was cured for 2 h at 95 °C. The channel was bonded by a cover glass (NEO cover glass, Matsunami Glass Ind., Osaka, Japan) using a plasma etching equipment (FA-1, Samco Inc., Kyoto, Japan). To prevent unintended contact between the droplets and the PDMS wall, we coated the surface of the microfluidic device with bovine serum albumin (BSA; A2153, Sigma-Aldrich).

### 2.3. Surface Tension Measurement

The surface tension of the droplets covered with the surfactant layer was measured using micropipette aspiration. The droplet size used in micropipette aspiration (~0.5 nL) was comparable to that generated by the microfluidic device. For the measurements, we derived the surface tension, *γ*, by:

∆*P* = *γ* (*R*_p_^−1^ − *R*_c_^−1^), (1)

where *R*_p_ and *R*_c_ are the inner radius of the pipette and the radius of a droplet, respectively, and *∆P* is the pressure difference between the inside and outside of the micropipette, which is monitored using a pressure transducer (DP15, Validyne, Northridge, CA, USA). The relaxation process of surfactant adsorption on the droplet surface was analyzed using the pendant drop method (DM-501, Kyowa Interface Science Co., Saitama, Japan).

### 2.4. Microscopic Observation of Droplets

Bright field images and fluorescence images of droplets were obtained by using an FV1200 confocal microscope (FV1200, Olympus, Tokyo, Japan). Temperature was controlled at ~25 °C using a temperature control system (TP-CH110RBF-C, Tokai Hit Co., Ltd., Shizuoka, Japan). As for fluorescence images of droplets containing calcein, the calcein was excited by a 473 nm laser and fluorescence signal was detected in the range of 490–590 nm.

### 2.5. Fluorescence Recovery after Photobleaching Experiment

To analyze the molecular transport of water-soluble ~5 μM calcein among the DIB-bounded droplets, we performed fluorescence recovery after photobleaching (FRAP) experiments using a confocal fluorescence microscope (FV1200, diffusion measurement package, Olympus, Tokyo, Japan). Calcein was transported through the reconstituted hemolysin at the DIBs of the droplets containing 62 nM α-hemolysin. The FRAP curve was obtained by illuminating a droplet within the honeycomb-patterned droplets. The illumination time was <1 s with a laser power of 100%. 

## 3. Results and Discussion

### 3.1. Microfluidic Device for Formation of Honeycomb-Patterned Droplets 

**Figure 2:**
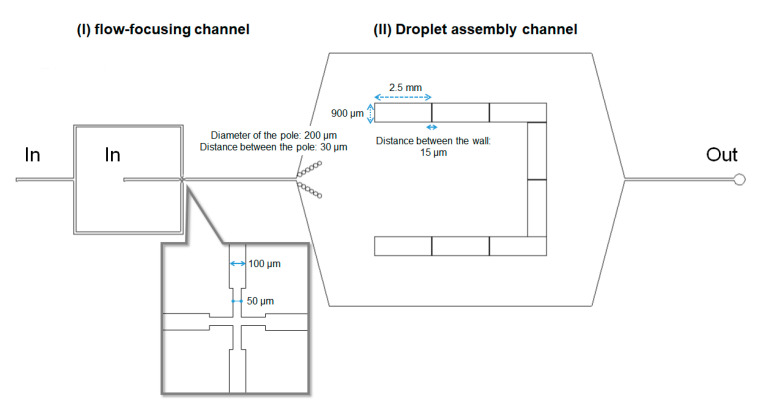
Schematic of the microfluidic device for spontaneous formation of honeycomb-patterned droplets. In section I, droplets were generated by a flow-focusing mechanism. In section II, the generated droplets were trapped and assembled in a U-shaped chamber.

To closely pack the droplets and form a honeycomb pattern in a 2D space, we designed a microfluidic device, as illustrated in Figure 2. The device included two sections: I and II. In section I, droplets were generated by a flow-focusing mechanism. In section II, the generated droplets were trapped and assembled in a U-shaped chamber. Chamber walls, 2.5 mm in width and 60 μm in height, were placed at 15 μm intervals to trap the droplets and remove the excessive oil. The dimensions of the flow-focusing channel and the droplet assembly channel were 50 μm in width and 60 μm in height. Flow rates for the oil phase (*Q*_o_) and the water phase (*Q*_w_) were changed in the range of 10–300 μL/h and 1–30 μL/h, respectively. The *Q*_w_/*Q*_o_ ratio was fixed at 0.1–0.25, where a droplet with a diameter of 20–60 μm was formed via jetting. As the height of the microfluidic device (i.e., 60 μm) was set to be comparable to the droplet diameter, the droplet arrangement was limited to 2D space. This condition allowed the droplets to be packed closely in 2D space.

### 3.2. Surfactant Condition for Honeycomb Pattern Formation of DIB-Bounded Droplets

To prepare a honeycomb pattern with DIB-bounded droplets, the droplets needed to form this pattern before droplet adhesion via DIB formation, because rapid DIB formation between the droplets prevents their spatial reordering in a microfluidic device. Therefore, we selected Span 80, which does not have any DIBs-forming ability. The adsorption rate of Span 80 on the droplet surface is much higher than that of the lipids, so the droplets were expected to form a honeycomb pattern prior to DIBs formation (Figure 1b).

**Figure 3:**
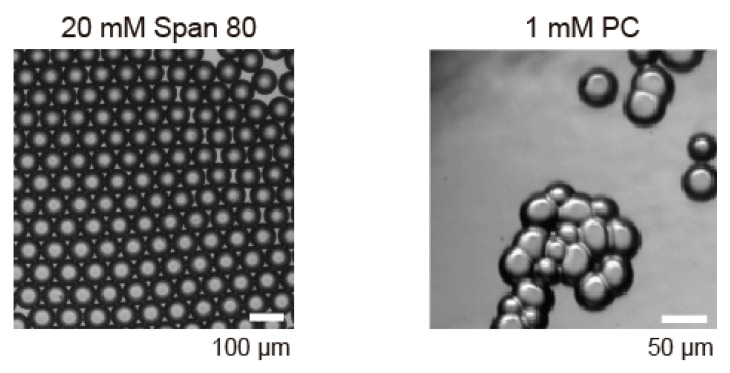
Microscopic images of droplets prepared in microfluidic device using oil with 20 mM Span 80 (**left**) and 1 mM PC (**right**). Flow rate ratio between water phase and oil phases, *Q*_w_/*Q*_o_, was fixed as 0.1 with *Q*_o_ = 300 μL/h.

Initially, we prepared droplets inside the microfluidic device using only Span 80 or the lipid PC as shown in bright field images (Figure 3). In the case of Span 80, each droplet was stably isolated and flowed to the U-shaped pool (section II in Figure 2) after the droplet formation in the flow-focusing channel (section I in Figure 2). As the number of droplets increased with time, the spatial rearrangement of the droplets progressed in the U-shaped pool, and finally formed a honeycomb pattern (Figure 3, left). A low concentration of Span 80 (<20 mM) was found to destabilize the droplets and initiate droplet coalescence. In comparison, droplets of 1 mM PC adhered to each other just after their contact and formed randomly arranged aggregates (Figure 3, right). Most PC droplets were unstable and coalesced on collision in the U-shaped pool. These results clearly showed the necessity of the lipid, PC, for DIB formation, which was in agreement with a previous study wherein bilayer vesicles (or liposomes) could not be formed without the addition of lipids to Span 80 [30]. 

**Figure 4:**
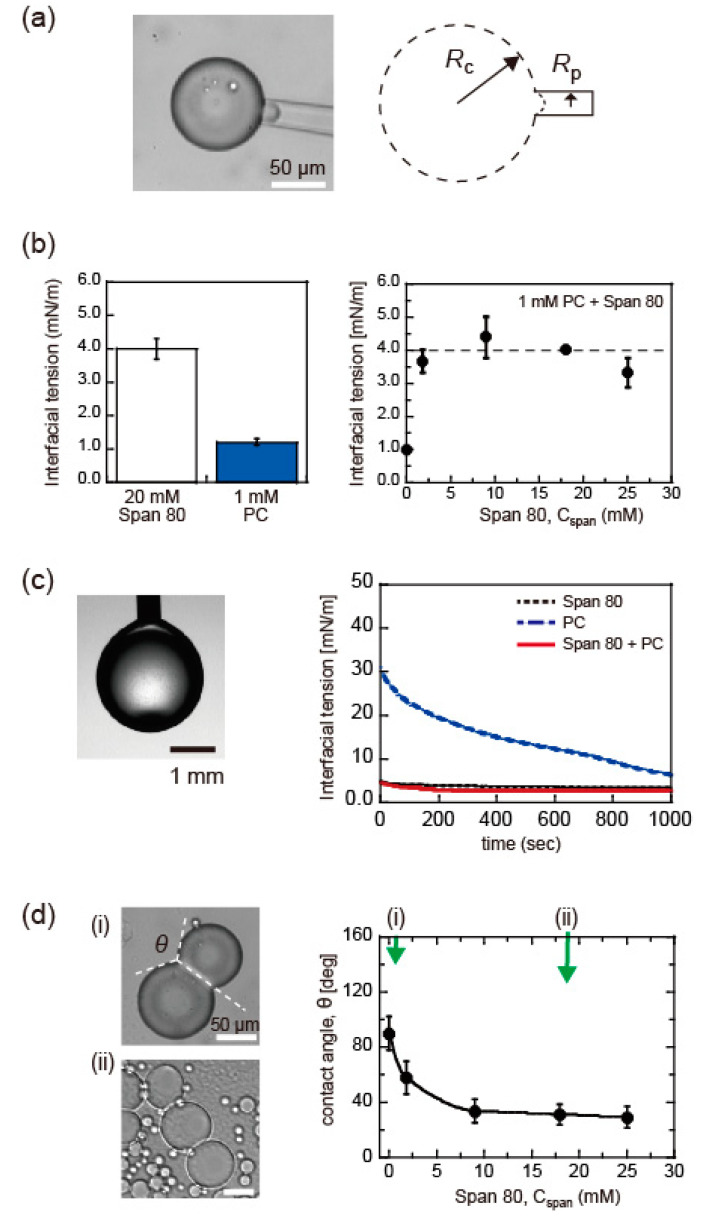
(**a**) Interfacial tension measurement by micropipette aspiration, where droplet radius *R*_c_ and inner radius of micropipette *R*_p_ are analyzed. (**b**) Values of interfacial tension (Average (Ave.). ± standard deviation (SD), *n* > 5) using micropipette aspiration for 20 mM Span 80 droplets, 1 mM PC droplets (left), and droplets with a mixture of 1 mM PC and various concentrations of Span 80, *C*_span_ (right). The broken line shows the value interfacial tension for 20 mM Span 80 (i.e., 4.0 mN/m). (**c**) Dynamic interfacial tension measurement using the pendant droplet method for 20 mM Span 80 (black dashed line), 1 mM PC (blue broken line), and their mixture (solid red line). (**d**) (left) Bright field images of DIB-bounded droplets for (i) 1 mM PC and (ii) a mixture of 1mM PC and ~18 mM Span 80, respectively. (right) Contact angle *θ* (Ave. ± SD, *n* > 5) for DIB-bounded droplets of a mixture of 1 mM PC and various concentrations of Span 80, *C*_span_.

Because the stability of the droplets is mainly determined by the surface tension, *γ*, at the water-in-oil droplet surface, we initially investigated *γ* for isolated droplets by micropipette aspiration (Figure 4a). In the case of 20 mM Span 80 and 1 mM PC, the values of *γ* are 4.0 ± 0.3 mN/m and 1.2 ± 0.1 mN/m (average (Ave.) ± SD), respectively (Figure 4b, left). These values were in agreement with previous reports [1,31]. Under a fixed concentration of 1 mM PC, we added a few concentrations of Span 80 (*C*_span_) and analyzed the change in *γ* (Figure 4b, right). The presence of Span 80 with *C*_span_ > 1 mM increased the value of *γ* similar to that of droplets with only Span 80 (i.e., ~4 mN/m). These results indicated that Span 80 adsorbs on the isolated droplet surface preferentially compared to the lipid PC.

To understand the surfactant adsorption process on the droplet surface, we subsequently analyzed the dynamic interfacial tension using the pendant droplet method. The time development of *γ* for an isolated droplet is shown for 20 mM Span 80, 1 mM PC, and their mixture in Figure 4c. The interfacial tension of Span 80 immediately reached a value of ~4 mN/m (black dashed line), which agreed with the corresponding *γ* value derived by the micropipette aspiration (Figure 4b, left). The addition of 1 mM PC to 20 mM Span 80 did not change the relaxation curve significantly (red solid line), and the values were similar to the *γ* values derived by micropipette aspiration (Figure 4b, right). Compared to these data, the relaxation rate of PC is remarkably low (blue broken line). The relaxation time of PC, derived from the exponential fitting, is approximately 700 s. the *γ* value did not relax to the value obtained by micropipette aspiration (i.e., ~1 mN/m; Figure 4b, left), which could reflect the difference in droplet size. These results demonstrated that the monolayer of Span 80 covering the droplet did not incorporate lipid PC, although the saturated *γ* value of PC was smaller than that of Span 80 (Figure 4b).

DIB-bounded droplets of the Span 80 and PC mixture were prepared by pipetting, as presented in Figure 4d. Contact angle *θ* between the adhesive droplets of PC was approximately 90°, as reported previously [1]. The addition of Span 80 decreased the contact angle to 30° with an increase in *C*_span_ from 0 to 25 mM (Figure 4d, right). This suggested that the surface-to-surface contact of the droplets initiated incorporation of the lipids in the contacted interface and accordingly led to DIB formation, unlike isolated droplets. However, the contact angle was smaller than the 120° required to form honeycomb-patterned droplets.

This tendency of the contact angle agreed with a previous report using Span 80 and lipids by Jeong et al., in which a droplet adhered to a flat interface covered with a surfactant monolayer and formed DIB between it and the contact interface [32]. They illustrated that Span 80 was removed from the contact interface during the DIB formation. Similarly, under our experimental conditions, the flowing out of Span 80 from the contact interface could support DIB formation when the droplets accidentally contacted each other during pipetting. When the droplets are generated by the microfluidic device, the microfluidic flow could initiate the replacement of Span 80 with PC and accordingly promote DIB formation. To validate this concept, we analyzed the pattern formation process of the droplets using the microfluidic device in the following experiments.

### 3.3. Honeycomb Pattern Formation of DIB-Bounded Droplets in Microfluidic Device

At a fixed concentration of 1 mM of lipid PC, we increased the concentration of Span 80. We found that the addition of ≥20 mM of Span 80 to 1 mM PC resulted in the spontaneous formation of a honeycomb pattern by the surface-contacted droplets. Therefore, in the following experiments, we fixed the composition as 1 mM PC and 20 mM Span 80 for preparing honeycomb-patterned droplets. In the case of *Q*_w_ ≈ 30 μL/h, droplets with a diameter 60 μm were produced at a rate of ~74 droplets/s. Because the size of the U-shaped region was very large (5.0 × 6.6 mm), it took a long time to fill the entire chamber with droplets. Therefore, we observed that a close-packed assembly consisting of hundreds of droplets formed near the wall after 7–10 min.

**Figure 5:**
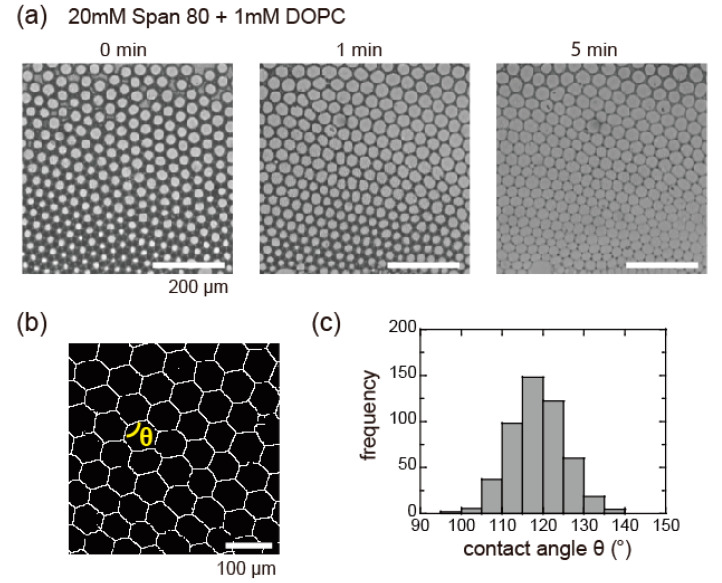
(**a**) Bright field images show the time evolution of hexagonal honeycomb-patterned droplets with DIBs from isolated droplets covered with monolayer of 1mM PC and 20 mM Span 80. Scale bar is 200 μm. (**b**) Binary image of honeycomb-patterned droplets. Scale bar is 100 μm. (**c**) Histogram of contact angle *θ* (119 ± 7, *n* = 497) derived from binary images of honeycomb-patterned droplets.

Figure 5a presents the time development of the closely packed droplets and the DIB formation in the U-shaped pool (section II in Figure 1b and Figure 2). With elapsing time, the oil between the droplets gradually decreased under the microfluidic flow. A few droplets began to adhere to adjacent droplets, and eventually all the droplets connected to each other via DIB formation (Figure 5a, right edge). The DIB formation between the droplets started from the upper right position and propagated throughout the droplets within approximately 7 min. We used a binary image of the obtained droplet assemblies (Figure 5b) to analyze the contact angle, *θ*, between the DIB-bounded droplets. Given that the histogram of *θ* (Figure 5c) had a peak at approximately 120°, the droplet assemblies were found to form a close-packed honeycomb pattern. These results supported our concept, i.e., that microfluidic flow initiates the replacement of Span 80 with PC and accordingly promotes DIB formation between adhered droplets. Relaxation of the surfactant molecules on both sides of the droplets were facilitated by the microfluidic flow, because the top and bottom of the droplets were attached to the glass cell, as suggested previously [33]. Under the experimental conditions, we obtained the adhesive interface profile in 2D. Acquiring the time evolution of the 3D profile would help to estimate the amount of oil remaining between the droplets and elucidate the mechanism of DIB formation.

### 3.4. Molecular Transport through DIB-Bounded Droplets via Reconstituted Nanopores

**Figure 6:**
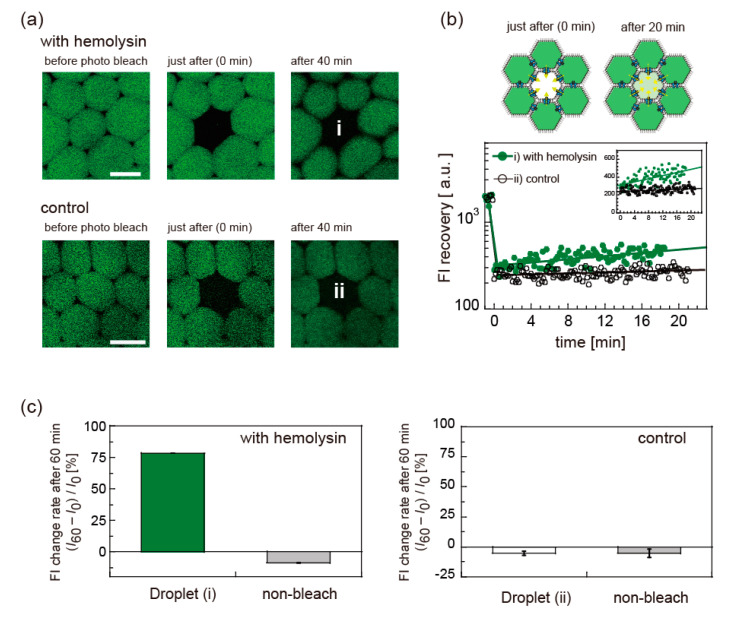
Fluorescence recovery after photobleaching (FRAP) experiments of adhered droplets containing water-soluble fluorescent solution (calcein, green) with or without hemolysin reconstituted into DIBs. (**a**) Confocal fluorescence images before and after photobleaching for DIB-bounded droplets with or without α-hemolysin (control). (**b**) (upper) Schematic of FRAP experiments where the droplet at center is photobleached. (lower) Fluorescence intensity (FI) change after photobleaching is shown as logarithmic scale and linear scale (inset). Solid lines are eye guides. (**c**) FI change rate within 60 min after photobleaching. (*I*_60_ − *I*_0_)/*I*_0_ where *I*_0_ and *I*_60_ are FI just after the photobleaching and after 60 min, derived from two different measurements is shown for photobleached droplets (droplets (i,ii) in Figure 6a) and non-photobleached droplets, respectively.

To examine the applicability of the DIB-bounded droplets to model cell tissues, we reconstituted a membrane protein nanopore of α-hemolysin into the DIBs between the droplets. We analyzed the water-soluble fluorescence transport of ~5 μM calcein. Because calcein is known to be almost impermeable to lipid bilayers [3] and the surfactant layer of Span 80 [34], calcein transport among the droplets ensured sufficient DIB stability to express the function of hemolysin. Figure 6 shows the fluorescence recovery experiments for the DIB-bounded droplets with or without hemolysin inside. After 20 min of photobleaching (<1 s), the fluorescence intensity (FI) was slightly recovered in the presence of hemolysin only (Figure 6a,b). FI change rate within 60 min after photobleaching, (*I*_60_ – *I*_0_)/*I*_0_ where *I*_0_ and *I*_60_ are FI just after the photobleaching and after 60 min, derived from two different measurements, shows the difference between calcein with and without the hemolysin (Figure 6c). Except the droplet (i) with hemolysin, all droplets had negative values, reflecting the fading of the fluorescent probe upon laser irradiation. Therefore, the experimental data supported that the reconstituted hemolysin at the DIBs successfully activated its transporting function. The reason for the low recovery rate of the FI could be related to the small amount of reconstituted hemolysin because non-ionic Span 80 is unlikely to induce hemolysin denaturation. When we increased the hemolysin concentration from 62 to 100 nM, we observed no apparent change in FRAP recovery rate. The addition of cholesterol and oleic acid could increase the recovery rate of the FI, as reported previously [35,36]. In addition, this FRAP experiment was particularly challenging because the laser irradiation triggered oil flow, destabilizing the droplets. We would like to address stability issues of the DIB-bounded droplets in the future.

## 4. Conclusions

In this study, we demonstrated the microfluidic formation of a honeycomb pattern of DIB-bounded droplets using a lipid, PC, and a surfactant, Span 80. Because Span 80 has a much higher adsorption rate than PC and no DIB formation ability, the droplets were immediately stabilized by Span 80 adsorption and spontaneously formed a close-packed honeycomb pattern in a thin microfluidic device. Under the microfluidic flow, Span 80 was replaced by PC, and the droplets adhered to each other via DIB formation. The DIB was sufficiently stable for membrane protein nanopores to exhibit the molecular transport function. This method, focusing on the adsorption rate, enabled the formation of a honeycomb pattern of DIB-bounded droplets without changing the microfluidic device design, and thus, introduced a novel aspect for droplet manipulation through the kinetic pathways of surfactant adsorption.
